# Computational Simulation of Scleral Buckling Surgery for Rhegmatogenous Retinal Detachment: On the Effect of the Band Size on the Myopization

**DOI:** 10.1155/2016/3578617

**Published:** 2016-06-20

**Authors:** Elena Lanchares, María A. del Buey, José A. Cristóbal, Begoña Calvo, Francisco J. Ascaso, Mauro Malvè

**Affiliations:** ^1^Aragón Institute of Engineering Research (I3A), University of Zaragoza, Campus Río Ebro, Calle Mariano Esquillor s/n, 50018 Zaragoza, Spain; ^2^Biomedical Research Center in Bioengineering, Biomaterials and Nanomedicine (CIBER-BBN), Aragón Health Sciences Institute, Calle Mariano Esquillor s/n, 50018 Zaragoza, Spain; ^3^Department of Ophthalmology, Hospital Clínico Universitario “Lozano Blesa”, Avenida San Juan Bosco 15, 50009 Zaragoza, Spain; ^4^Department of Surgery, School of Medicine, University of Zaragoza, Domingo Miral s/n, 50009 Zaragoza, Spain; ^5^Aragón Health Research Institute (IIS Aragón), Aragón Biomedical Research Center (CIBA), Avenida San Juan Bosco 13, 50009 Zaragoza, Spain; ^6^Department of Mechanical Engineering, Energetics and Materials, Public University of Navarra, Campus Arrosadía, 31006 Pamplona, Spain

## Abstract

A finite element model (FE) of the eye including cornea, sclera, crystalline lens, and ciliary body was created to analyze the influence of the silicone encircling bandwidth and the tightness degree on the myopia induced by scleral buckling (SB) procedure for rhegmatogenous retinal detachment. Intraocular pressure (IOP) was applied to the reference geometry of the FE model and then SB surgery was simulated with encircling bandwidths of 1, 2, and 2.5 mm. Different levels of tightening and three values of IOP were applied. The anterior segment resulted as unaffected by the surgery. The highest value of Cauchy stress appeared in the surroundings of the implant, whereas no increment of stress was observed either in anterior segment or in the optic nerve head. The initial IOP did not appear to play any role in the induced myopia. The wider the band, the greater the induced myopia: 0.44, 0.88, and 1.07 diopters (D) for the 1, 2, and 2.5 mm bandwidth, respectively. Therefore, patients become more myopic with a wider encircling element. The proposed simulations allow determining the effect of the bandwidth or the tightness degree on the axial lengthening, thus predicting the myopic increment caused by the encircling surgery.

## 1. Introduction

The conventional surgical treatment for rhegmatogenous retinal detachment (RRD) is 360-degree scleral buckling (SB). It is an effective procedure to achieve retinal reattachment and has been used over 60 years [1, 2]. Since the introduction of* pars plana* vitrectomy (PPV) in 1971 [3], the surgical treatment of RRD consists of these two procedures: encircling procedure and posterior* pars plana* vitrectomy, either alone or combined.

The SB surgery begins with the application of locoregional or, in some cases, general anesthesia. After having hooked the eye muscles using silk surgical thread, the encircling silicone band is guided under the muscle insertions. The encircling element is secured to the sclera 12-13 mm posterior to the limbus with monofilament nylon mattress sutures placed in the four quadrants between the muscles. The encircling implant is fastened with a sleeve with moderate tightness and the surplus end is cut from the band. Then, PPV would be performed if it was planned.

The increment of myopic defect observed in eyes that underwent encircling scleral buckle is due to an increase of the anteroposterior axial length caused by circumferential indentation. Some works in the literature [4, 5] found that the encircling band surgery does not modify the anterior segment of the eye and therefore the degree of myopia induced during the surgery would be exclusively due to the lengthening of the posterior segment of the eye. Although other authors observed a significant modification of the anterior chamber depth after surgery [6, 7], recovery of its normal value in 9–12 months was reported [7].

Finite element (FE) models will help to predict the surgical results and determine the influential parameters [8–14]. Numerical models of the eye have been used to analyze the mechanical response of the eye to different ocular surgeries such as arcuate and limbal relaxing incisions for astigmatism correction, photorefractive keratectomy for the correction of myopia [8–10], and specifically scleral buckling (SB) surgery [11–14]. Before the SB surgery, the size of the encircling band and the position and pull tightening must be established. The more tightened the band, the more the possibilities of bringing the retinal pigment epithelium in contact with the detached sensory retina. Nevertheless, the increment of the axial length will be greater, and thus it will induce higher levels of myopia.

Idealized numerical models of the eye created from average dimensions provide qualitative information on the response of the eye to surgery [8, 15]. Patient specific models would supply quantitative results applicable to a specific patient [16, 17]. In both cases, the accuracy of results will depend on the quality of the model, which must reproduce the geometry of the eye and the mechanical behavior of the tissues.

This work is not intended to simulate retinal attachment achieved by the SB surgery. Assuming that the operation is successful, the aim of this study is to investigate, by means of a numerical model of the eye, the degree of myopia induced by the SB surgery according to the width and the tightening of the encircling element, in order to minimize this collateral effect.

In particular, the scope of this work is to predict and quantify the effect of the main parameters related to the scleral buckling. For this reason, a numerical model based on average data of a large number of patients is provided. The proposed investigation is more parametric than patient specific. The advantage of this numerical model of the eye is that it is possible to perform many variations of the different parameters without altering the patient functionalities and considerably reducing the animal experimental tests.

We simulated two commonly employed bandwidths: 2.0 mm and 2.5 mm, respectively. Besides these two sizes, we also used a 1 mm width to analyze the efficiency of such a narrow band, since it may induce lower myopia increment. Three values of IOP (11, 15, and 18 mmHg) were considered.

## 2. Materials and Methods

### 2.1. Finite Element Model

A spherical three-dimensional finite element model of the eyeball was created using Rhinoceros v.4.0 (McNeel & Associates, Indianapolis, IN, USA). This idealized model is based on average eye dimensions and was validated with other surgical techniques in previous works [8–10].

**Figure 1:**
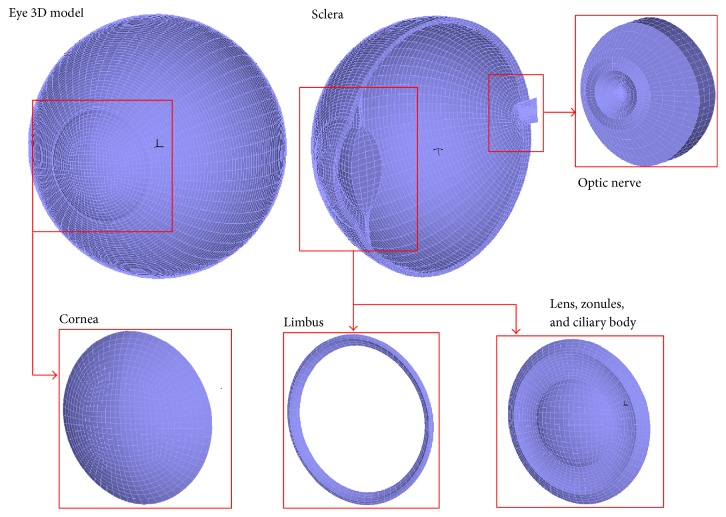
Finite element model of the eye. Components of the model used in this work for the simulation of the scleral buckling surgery.

Figure 1 shows the parts of the model, namely, cornea, limbus, sclera, lens, zonules, and ciliary body and the optic nerve. The model of the cornea was based on a nonrevolution ellipsoid with semiprincipal axes of lengths *a* = 10.43, *b* = 10.27, and *c* = 14.26 mm [18] with a sagittal corneal diameter of 11.26 mm and transversal diameter of 11.51 mm; the central corneal thickness was 550 microns. The crystalline lens had an anterior radius of 11 mm, while the posterior radius was −6.5 mm. The eyeball was modelled with a nonspherical geometry, whose dimensions were 25.43 mm (sagittal), 25.76 (transverse), and 24.86 mm (axial). The thickness of the sclera varies with the distance to the limbus and was modelled following Olsen et al. [19].

The created geometry was meshed using the commercial software Ansys ICEM CFD v.14.5 (Ansys Inc., Canonsburg, PA, USA). The numerical model, composed of 34430 nodes, was obtained after an appropriate grid sensitivity analysis. This independence study was conducted comparing the displacements obtained under the same conditions to different mesh refinements.

The human eye is composed of porous tissues with high water content. Approximately 80 percent of the corneal weight is due to water. Cornea and sclera are composed of long collagen fibres embedded in a ground substance mainly formed of proteoglycans and water. In the cornea, collagen fibres lie parallel and run along the whole length of the lamella. In the central region of the cornea, fibres are orthogonally disposed along the superior-inferior and nasal-temporal directions whereas they are predominantly circumferential near the limbus. Moreover, there are other randomly oriented fibres throughout the cornea. This microstructure and the different distributions of collagen fibres give the corneal tissue an anisotropic mechanical behavior [20]. According to this, the cornea was considered as an anisotropic hyperelastic material with two preferred material directions. The two families of fibre directions were as follows: one along the nasal-temporal direction and the other one along the superior-inferior direction (see Lanchares et al. [8]). A circumferential direction of collagen fibres was defined in the limbus, whose material parameters have been assumed identical as for the cornea. The sclera was considered as an isotropic material [21].

The human crystalline lens is composed of nucleus, cortex, and capsule. The fibres in the nucleus are not clearly arranged; therefore the nucleus is considered as an isotropic material. In spite of the clear arrangement of the fibres in the cortex [22], it was modelled as an isotropic material due to the lack of data for the specific material parameters. Isotropy was also assumed for the neural tissue of the optic nerve. The capsular tissue of the lens is increasingly stiffer circumferentially towards the equator [23]; therefore a preferential circumferential direction of deformation was considered.

An appropriate strain energy density function Ψ is required from which stress-strain relations and local elasticity tensors are derived [24]. Because of the directional dependence on the deformation of the tissues, we use a unique decoupled representation of Ψ [25] which depends on the right Cauchy-Green tensor **C** = **F** ^*T*^ **F** with **F** being the deformation gradient tensor. To represent the dependence of Ψ on the directions of the fibres in the reference configuration **m**_0_ and **n**_0_, the structural tensors **M** = **m**_0_ ⊗ **m**_0_ and **N** = **n**_0_ ⊗ **n**_0_ are used:

(1) $$\begin{array}{c} \Psi \left(\;C\;\right)=\Psi_{\text{vol}}\left(\;J\;\right)+\bar{\Psi }\left(\;\bar{C},M,N\;\right), \end{array}$$

where Ψ_vol_(*J*) characterizes the change in volume and $\bar{\Psi }\left(\bar{C},M,N\right)$ characterizes the change in shape, with *J* = det⁡(**F**) and $\bar{C}={\bar{F}}^{T}\bar{F}$ the modified right Cauchy-Green tensor, with ${\bar{F}=J}^{-1/3}F$.

Then, we use the following strain energy function Ψ:

(2) ΨΨvol+Ψ¯matrix+Ψ¯fibres=12Dln⁡J2+C12I1¯−3+C22I2¯−3+k12k2exp⁡k2I4¯−12−1+k32k4exp⁡k4I6¯−12−1,

where 1/*D* is a penalty coefficient for numerical purposes; ${\bar{\Psi }}_{matrix}^{}$ corresponds to the isochoric change of the matrix of the tissue; and ${\bar{\Psi }}_{fibres}^{}$ represents the isochoric change due to the fibres in the tissue. $\bar{I_{1}}$ and $\bar{I_{2}}$ are the first two modified strain invariants of the symmetric modified right Cauchy-Green tensor $\bar{C}$. The pseudoinvariants $\bar{I_{4}}$ and $\bar{I_{6}}\;$characterize the constitutive response of the fibres. They have a clear physical meaning, the square of the stretch *λ* along the fibre directions [26]. *C*_1_, *C*_2_, *k*_1_, *k*_2_, *k*_3_, and *k*_4_ are the parameters of the model that characterize each tissue. These material properties of the tissues were taken from several authors [8, 27–29] and are listed in Table 1.

### 2.2. Simulation of the Surgery and Analysis of Results

The process of simulation was performed using the commercial software Adina v8.5 (ADINA R&D Inc., Watertown, MA, USA). To set the model in physiological conditions, the dimensions must reproduce those measured of the eye* in vivo*. Nevertheless, such geometry belongs to a deformed configuration of the eye due to the effect of the IOP but the tissue prestress is neglected in the model. Consequently, in a first step, both the boundary conditions and the physiological internal stress distribution must be introduced in the FE model in order to balance the IOP. An iterative process was then used to incorporate into the model the initial strains by means of the deformation gradient **F**_0_^*n*^ = **F**_*n*−1_^*n*^ **F**_0_^*n*−1^ which balances the IOP. At the end of the process, the final configuration of the model matches the initial one. This methodology is explained in detail in Lanchares et al. [8].

**Figure 2:**
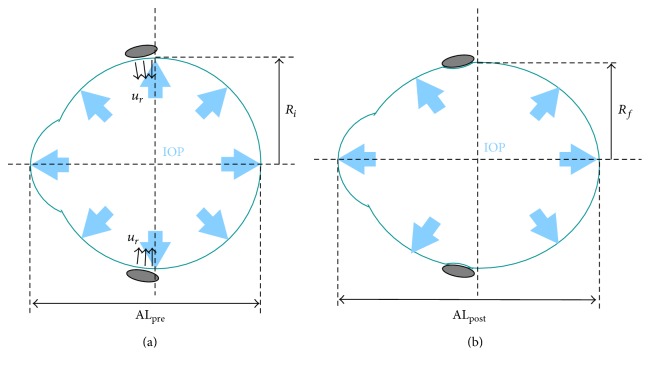
Eyeball before (a) and after (b) encircling scleral buckling surgery. The light-blue bold arrows represent the IOP acting on the inner surface of the eye. *R*_*i*_ is the radius of the eyeball at the position where the band is planned to be attached, *u*_*r*_ (line arrows) is the radial displacement towards the inner of the eye at the tightening of the band, AL_pre_ is the axial length of the eye before the surgery, *R*_*f*_ is the radius of the sclera under the band after the implantation, and AL_pos_ is the postsurgical axial length.

In a second step we resolved the surgery. The simulation of the band implantation consists of the imposition of a given value of displacement in the negative radial direction (inwards) of the nodes positioned at the equator of the eyeball where a band of that width would be implanted (12-13 mm posterior to the limbus) causing elongation of the eye (Figure 2). The value of displacement *u*_*r*_ is calculated according to the tightening pressure to be applied to the band: *u*_*r*_ = *R*_*f*_ − *R*_*i*_, where *R*_*i*_ is the radius of the eyeball in the initial model at the meridian where the band is going to be implanted, before any simulation, and *R*_*f*_ is the radius of the eyeball under the band after the surgery, obtained from 2*πR*_*i*_ − 2*πR*_*f*_ = 10 mm, where 10 mm is the length of the surplus end cut from the band. The bands considered for this study were Mira silicone band-40 (2.0 mm wide and 0.75 mm thick) and Mira silicone band-240 (2.5 and 0.60 mm, resp.) (Mira, Inc., Waltham, MA, USA). Three values of IOP, 11, 15, and 18 mmHg, were applied to evaluate any influence of IOP on the final result.

To determine the change in myopia caused by the SB surgery, the process described by Wang et al. [12] was followed. The initial dioptric power of the eye (*D*_0_) is calculated according to the theoretical eye model, which considers the eye as a lens system composed of cornea (*D*_1_) and crystalline lens (*D*_2_): *D*_0_ = *D*_1_ + *D*_2_ − *D*_1_ *D*_2_ *d*/*n*_1_, where *d* is the distance between the anterior corneal apex and the middle surface of the crystalline lens and *n*_1_ is the corneal refractive index. Its value is 1.376, but, given the refractive indexes of the aqueous humor (1.336) and the tear film covering the anterior surface of the cornea (1.337), and since the cornea is very thin (0.55 mm thick), it is reasonable to simplify this optical system and consider only the curvature of the anterior surface and the refractive index of 1.336 for the refractive power of the cornea [12].

The degree of myopia induced by the surgery (Δ*D*) is obtained as the subtraction of the final refraction of the eye (*D*_0*f*_) from the initial refraction (*D*_0_): Δ*D* = *D*_0_ − *D*_0*f*_ = *D*_0_ − (*n*_1_/AL), where AL is the postoperative axial length of the model.

## 3. Results

**Figure 3:**
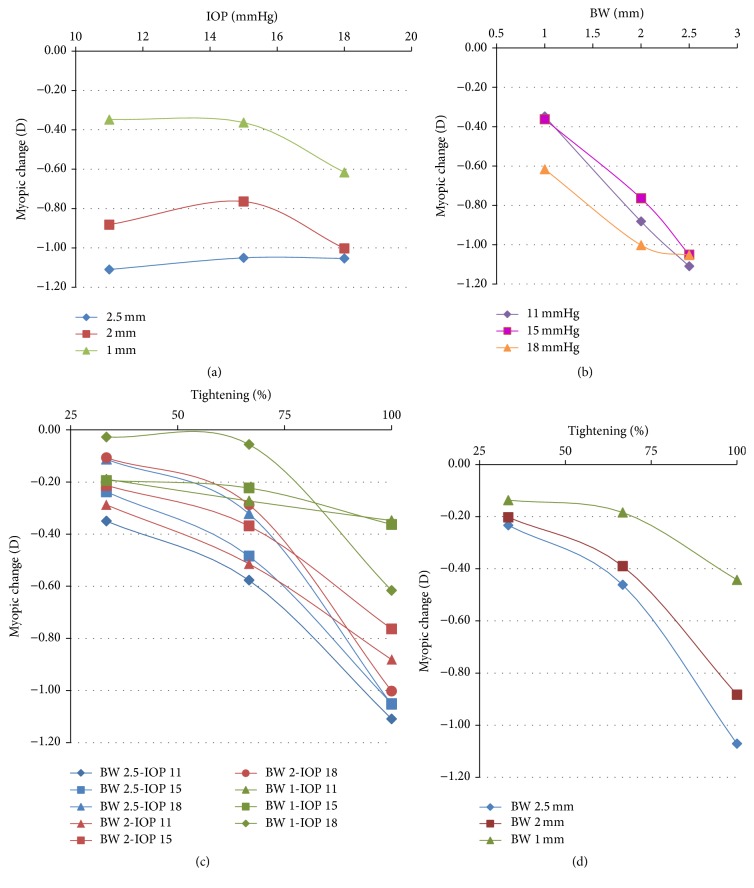
Influence of (a) IOP (mmHg) and (b) BW (mm) on the myopia induced, in diopters (D). No clear tendency of the induced myopia is observed with increasing IOP. On the contrary, bandwidth has a role in the postsurgical myopia change. The wider the band is, the greater the degree of myopia is induced. (c) Myopic changes obtained for all the simulated cases. (d) Mean values of the myopic change induced by the three bandwidths considered: 1, 2, and 2.5 mm.

Figure 3(a) shows the line graph of myopia induced* versus* IOP. With increasing IOP, each band shows a different behavior. While the 2 mm wide band has a lower effect at 15 mmHg than at 11 mmHg, the 1 mm wide band shows a slight increase in its effect between these two values of IOP; both of them achieve a higher myopic change at 18 mmHg. The 2.5 mm wide band induces approximately the same myopic change for the three values of IOP. Thus, no clear trend can be inferred. The graph in Figure 3(b) represents the myopic change obtained by numerical simulation* versus* the bandwidth for the three values of IOP considered. Although only the 2.5 mm wide band gives the same results for the three values of IOP, no trend can be inferred from the results corresponding to the other two bandwidths. Thus, we assumed no relationship between myopic change and IOP, so the myopic changes obtained by numerical simulation were averaged for all the three values of IOP (11, 15, and 18 mmHg).

The modification of the axial length and myopic change after the simulation of the surgery for all the cases considered are compiled in Table 2. The mean axial length increment was 0.21 ± 0.07, 0.42 ± 0.06, and 0.51 ± 0.01 mm for the 1.0, 2.0, and 2.5 mm wide bands, respectively. The mean myopic increment for the 1.0, 2.0, and 2.5 mm wide bands was −0.44 ± 0.15, −0.88 ± 0.12, and −1.07 ± 0.03 D, respectively.

Table 3 shows the increment of myopia caused by three levels of tightening of the encircling band,* that is,* 33%, 67%, and 100%, of the final tightening for all nine cases (see Figure 3(c)). In agreement with the assumption made above, no correlation of the induced myopia with IOP was observed. Thus, the resulting data obtained for the three bandwidths and the three values of IOP at each level of tightening were averaged and included in Table 3 and depicted in Figure 3(d). The 1 mm wide band had a considerably lower effect than the 2.0 and 2.5 mm bands. For all the bandwidths considered, the observed trend predicts substantially greater increments of induced myopia for levels of tightening over 100%. In order to estimate these increments, we used a power function of the form *a∗x* ^*b*^ + *c* that fits the outcoming data, where *x* represents the percentage of tightening applied to the encircling band. Using Matlab R2013a (The MathWorks, Natick, MA, USA), we obtained the following functions for the three bandwidths: −4.80 · 10^−11^ · *x* ^4.899^ − 0.1387 (1 mm), −1.12 · 10^−06^ · *x* ^2.901^ − 0.1716 (2 mm), and −1.051 · 10^−06^ · *x* ^2.96^ − 0.1972 (2.5 mm). Changes of −1.04, −1.52, and −1.89 D were obtained, respectively, for a tightening of 125%. That means that an increment of 25% in the tightening of the band causes increments in myopia of 136%, 73%, and 76% for the 1, 2, and 2.5 mm wide bands, respectively.

**Figure 4:**
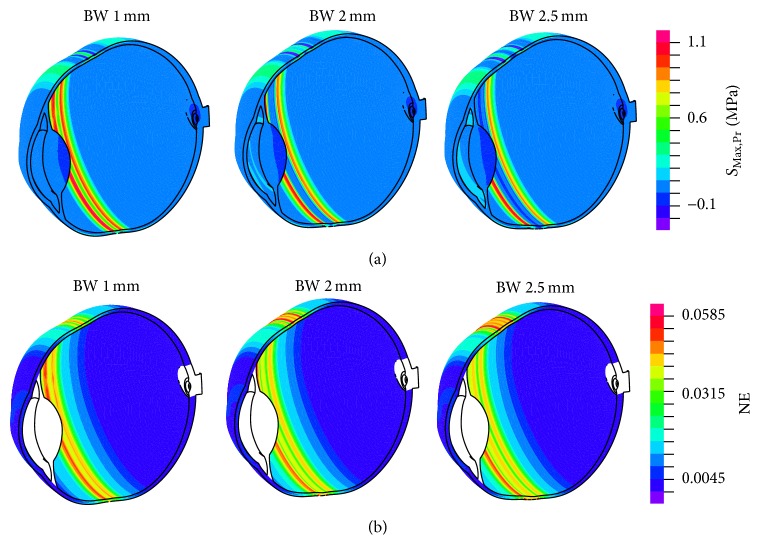
Stress and strain distribution in the model after surgery. The three upper images show the maximal principal stress (*S*_Max,Pr_) distribution (in MPa) in the model of the eye after the simulation of 1, 2, and 2.5 mm wide bands at an IOP of 15 mmHg. The images at the bottom show the logarithmic strain NE (nondimensional) in the model after the simulation of the surgery for the same three cases.

Figure 4 shows the maximal principal stress distribution and the logarithmic strain in the model (15 mmHg) following the simulation of surgery, for the three bandwidths (1.0, 2.0, and 2.5 mm). The highest values of both parameters appear in the area where the band was implanted. Neither the anterior segment nor the optic nerve head area was affected by the simulated surgery.

The anterior segment of the eye model was not altered by the SB simulation in any of the cases since no change was observed in corneal refraction, corneal thickness, and anterior chamber depth.

## 4. Discussion

Numerical simulation of blunt trauma causing retinal detachment has been accomplished by some authors [30, 31] as well as scleral buckling surgery either from a mechanical approach or by coupling fluid mechanics with structural mechanics [12–14]. Kim et al. [14] simulated encircling SB surgery with a spherical FE model of the eye composed of two layers, outer sclera and inner choroid-retina, using a linear elastic constitutive model to characterize the tissues. Wang et al. [12] studied the effect of segmental SB surgery with FE model of the eye that included the vitreous humor. All the tissues were modelled as hyperelastic materials but considered isotropic. In this work, we introduced anisotropy in those parts of the FE corresponding to tissues with fibres preferentially aligned in given directions [8, 27].

The encircling scleral buckling procedure enlarges the axial length of the eye, resulting in an increment of myopia, which has been the object of the present study. A finite element model of the eye was developed to evaluate the myopic effect of this technique by means of numerical simulation. The purpose is to present a numerical tool to analyze the encircling SB surgery in a numerical approach for predicting its effect on the human eye before the surgery. All the parameters involved, such as bandwidth, IOP value, and tightening of the encircling silicone element, can be modified and their effects can be analyzed, while the eye features remain invariable. For this reason, a parametric model is necessary to firstly assess the effect of each variation on the human eye. This type of model has the advantage that many variations can be performed without compromising the patient functionality. This would also considerably reduce the number of experimental animal models.

In a future step, once clinically validated, the numerical simulations could be applied to a patient specific model for predicting the effect of the surgery on a specific subject before performing the encircling. This validation is crucial before the numerical tool can be accepted and introduced in the daily clinical praxis. However, this validation is not provided in this work because it is out of the aim of the study, which is oriented to providing qualitative results of the SB surgery.

Our conclusions must be considered from a qualitative point of view. If particular values were required, then a patient specific model should be developed, which entails an accurate modelling of both the geometry and the tissue mechanical response. In the clinical practice, each eyeball presents different dimensions; therefore the same values of the treatment parameters (bandwidth, etc.) induce a different degree of myopia on each patient. Those results obtained by numerical simulation can help the ocular surgeon to choose the optimal parameters while planning the surgery.

However, it has to be noted that different surgeons normally used different techniques when performing ocular surgery. For this reason, the application in the clinics of the results coming from the numerical simulations, which may provide a kind of numerical tool for surgical planning, may strongly depend on the surgeon and on the variability of the surgical technique. In this sense, the results presented in this work have to be considered as a first step into the future application of numerical techniques for clinical scopes. Considering that some surgeries are nowadays standard, we can reasonably assume this point as limitation of this study.

The numerical simulation showed no significant change in the anterior segment morphology (corneal curvature, corneal thickness, and anterior chamber depth) following the surgery. Therefore, the anterior segment resulted as unaltered after the simulation of SB. This conclusion is consistent with the clinical findings of previous studies in the literature [4, 5]. Other authors found a significant modification of the anterior chamber depth after the encircling band surgery [6, 7] but reported that the anterior chamber depth returned to normal at 1 year after surgery [7].

We analyzed the influence of silicone bandwidth, tightening degree, and IOP value on the myopic increment induced by the SB surgery. Regarding the bandwidth, the widest band (2.5 mm) caused the highest increment in myopia (1.07 D); hence we can conclude that the bandwidth plays a relevant role in the final myopia. The induced myopia due to the axial length increment is directly related to the width of the band. Moreover, other studies also found a strong relation with the thickness of the encircling silicone element [14], which we did not consider in our study.

We used a noncommercial 1 mm wide band to analyze the effect of the bandwidth on the eyeball lengthening. As expected, axial length was increased while the anterior segment dimensions remained intact. Nevertheless, the stress distribution in the scleral tissue next to the band was acutely concentrated, causing local effects which may lead to scleral tissue damage. This effect observed in the numerical model after the SB simulation reproduces a described complication of the technique pioneered by Arruga [32] who performed equatorial cerclage with a nylon, silk, or supramid suture, which in some patients produced a necrosis of the scleral tissue leading to intrusion of the suture into the eye [33].

The effect of the tightening of different bandwidths on the myopia change induced was also analyzed. Figure 3(d) shows a nonlinear relation between the tightening level and the increment of myopia induced. At 75% of the total tightening, the level of induced myopia is lower than half of the value of myopia at 100% tightening. The fitting function predicts considerable increments of induced myopia for levels of tightening over 100%, for all the bandwidths considered. An increment of 25% in the tightening of the band causes increments in myopia of more than 70% in all the three cases considered. From a clinical point of view, our results suggest that the surgeon should avoid overtightening of the implant. Moreover, it may also affect the anterior segment of the eye. These considerations should be validated with clinical studies.

Regarding IOP, we found no relation of the increment in myopia with this parameter. The variations considered here for the IOP (from 11 to 18 mmHg), that is, 7 mmHg, have shown to be negligible for this study. This result is consistent with Wang et al. [12] who observed a minimal effect of IOP values on the stress of the tissue following surgery. The values of IOP evaluated in our cases of simulation correspond to typical values in case of rhegmatogenous retinal detachment, which is usually preoperatively decreased. The IOP variation caused by the surgery cannot be measured by the numerical simulation process since the IOP value is an input parameter for the model.

With respect to the level of myopia induced by the surgery, the maximal value of myopia change in this study was obtained for the 2.5 mm wide band and IOP of 11 mmHg. In that case, the axial length was enlarged 0.52 mm corresponding to an increment of myopia of −1.1 D. In order to assess these outcomes, we only found a similar work in the literature (Kim et al. [14]). In contrast to our results, their model showed modification of the corneal curvature as well as shortening of the optical length of the eye; therefore the refractive error was modified following surgery. These results, opposite to ours, may be due to the absence of crystalline lens and ciliary body in their model, thus making it weaker. Moreover, the difference in the initial geometry of the model (they considered a spherical shape of the eye) may also cause differences between the two works. However, as it was explained above, the postsurgical values supplied by the simulation with our model correspond to the dimensions assigned to the preoperative model, and patient specific values would give more accurate results. The same value of inner radial displacement in a smaller eyeball would cause a greater effect since it would mean a greater tightening of the band. Kim et al. [14] reached the same conclusion in their study when the eyeball size was reduced by 15%. They observed an increment in the maximum stress in the sclera with respect to the reference size model under the same value of indentation force and IOP.

An important limitation of the presented work is that additional clinical studies are necessary to validate the refractive changes predicted by our model. The increment in myopia found here is slightly lower than the clinical outcome obtained by Smiddy et al. [34] in 75 eyes whose average induced myopia was 2.75 D with an average increased axial length of 0.99 mm. Some of these eyes were also implanted with a radial element, which would increase the myopia induced by the encircling surgery. Goezinne et al. [7] reported in a clinical study with 38 eyes a myopic change of 2.6 D for an axial length increment of 0.7 mm. This greater effect of the surgery on the induced myopia may be due to the use of a radial or segmental buckle associated with the encircling band in the reported cases. To achieve clinical validation of our results, patient specific models will help in the future, but it is out of the aim of this paper.

We also analyzed the stress distribution in the tissue after the surgery. The maximal level of stress obtained in our study is considerably lower than that considered to cause tissue damage. We obtained a maximal value of stress of 1.1 MPa in the most unfavorable of the cases (2.5 mm wide band with an IOP of 18 mmHg). According to Uchio et al. [35], the failure of the scleral tissue happens at a 6.8% strain, corresponding to a stress value of 9.5 MPa, and the linear elastic behavior ends at about 6.6 MPa. That means that between both values the tissue is damaged. Therefore, the stress value after the surgery is considerably lower than the elastic limit of the sclera, which means that no damage was caused.

One obstacle to overcome when modelling the eyeball is deciding the boundary conditions to impose. In this work, both the anterior segment and the optic nerve insertion of the model were free to move, whereas the eyeball in physiological conditions is fitted into the socket and surrounded by tissues in such a way that backward movement would be almost negligible. If we reproduce that situation in our model, that is, the backward movement set to zero, then the stress distribution in the retinal area computed by simulation would give unreasonable maximal values of stress, which would mean an overestimation of the damage caused by the SB surgery. Since this work was not intended to study the squashing of the back of the eye or the optical nerve, we assumed this as limitation of the model. Wang et al. [12] constrained the exterior posterior wall of the eyeball, nevertheless, they did not analyze the stress in the optical nerve head area but in the surroundings of the encircling implant, and hence the local effect of this boundary condition did not affect their area of interest.

In this study, we presented a numerical tool to estimate the myopic effect of the SB surgery on the human eye. In this sense we provided qualitative results based on different parameters of the encircling procedure. For an exact quantification of the myopic effect, a clinical validation based on clinical cases would be necessary to contrast the provided results. In particular, the design of models based on patient specific geometries instead of parametric values such as those used in this work will help improving the quality of the results and will help assessing the reliability of the clinical outcomes.

## Figures and Tables

**Table 1:** Material parameters of the tissues in the anisotropic fibred model of the eye. *D* is a penalty coefficient for computational purposes; *C*_1_ and *C*_2_ are the material parameters of the extracellular matrix. The parameters *k*_1_ and *k*_2_, *k*_3_, and *k*_4_ correspond to the two families of fibre directions, respectively.

Tissue	*D* (MPa^−1^)	*C*_1_ (MPa)	*C*_2_ (MPa)	*k*_1_ (MPa)	*k*_2_	*k*_3_ (MPa)	*k*_4_
Cornea	10^−5^	10^−1^	0	0.234	29.917	23.4 × 10^−2^	29.917
Limbus	10^−5^	10^−1^	0	0.234	29.917	0	0
Sclera	10^−5^	35	−32	0	0	0	0
Lens cortex	34.54	58.295 × 10^−5^	0	0	0	0	0
Lens nucleus	214.96	93.667 × 10^−6^	0	0	0	0	0
Lens capsule	28.35 × 10^−2^	21.60 × 10^−2^	0	3.39 × 10^−2^	9.7406	0	0
Nerve	4	50.335 × 10^−4^	0	0	0	0	0

**Table 2:** Increment of myopia (Δ*D*) in diopters (D) induced by the scleral buckling surgery in the numerical model. Nine cases are considered. IOP is the intraocular pressure, BW is the width of the band implanted, AL_pre_ is the axial length before the simulation of the surgery, AL_pos_ is the axial length after the simulation, and ΔAL is the increment of axial length. The mean value of ΔAL and Δ*D* for each of the three bandwidths is also shown.

Case	IOP (mmHg)	BW (mm)	AL_pre_ (mm)	AL_pos_ (mm)	ΔAL (mm)	Δ*D* (D)
1	11	2.5	24.860	25.382	0.522	−1.11
2	15	2.5	24.860	25.356	0.496	−1.05
3	18	2.5	24.860	25.359	0.499	−1.06

Mean ± SD		2.5	—	—	0.51 ± 0.01	−1.07 ± 0.03

4	11	2	24.860	25.273	0.413	−0.88
5	15	2	24.860	25.219	0.359	−0.76
6	18	2	24.860	25.334	0.474	−1.01

Mean ± SD		2	—	—	0.42 ± 0.06	−0.88 ± 0.12

7	11	1	24.860	25.022	0.162	−0.35
8	15	1	24.860	25.029	0.169	−0.36
9	18	1	24.860	25.149	0.289	−0.62

Mean ± SD		1	—	—	0.21 ± 0.07	−0.44 ± 0.15

**Table 3:** Increment of myopia (Δ*D*) in diopters (D) induced by the scleral buckling surgery in the numerical model for the nine cases considered (bandwidths of 1, 2, and 2.5 mm; IOP of 11, 15, and 18 mmHg) at three different levels of tightening (33%, 67%, and 100%). Since no relation is observed with IOP, the mean value of the myopia induced for each of the three bandwidths is also shown.

Case	IOP (mmHg)	BW (mm)	Δ*D* (D) 33%	Δ*D* (D) 67%	Δ*D* (D) 100%
1	11	2.5	−0.35	−0.58	-1.11
2	15	2.5	−0.24	−0.48	-1.05
3	18	2.5	−0.11	−0.32	-1.06

Mean ± SD		2.5	−0.23 ± 0.12	−0.46 ± 0.13	−1.07 ± 0.03

4	11	2	−0.29	−0.51	-0.88
5	15	2	−0.21	−0.37	-0.76
6	18	2	−0.11	−0.29	-1.01

Mean ± SD		2	−0.20 ± 0.09	−0.39 ± 0.11	−0.88 ± 0.12

7	11	1	−0.19	−0.27	-0.35
8	15	1	−0.19	−0.22	-0.36
9	18	1	−0.03	−0.06	-0.62

Mean ± SD		1	−0.14 ± 0.09	−0.18 ± 0.11	−0.44 ± 0.15

## Abbreviations

FE: Finite element
SB: Scleral buckling
IOP: Intraocular pressure
RRD: Rhegmatogenous retinal detachment
PPV: * Pars plana* vitrectomy.
